# Antipsychotic-Induced Ogilvie Syndrome: A Case Report

**DOI:** 10.7759/cureus.80910

**Published:** 2025-03-20

**Authors:** José Ribeiro, Rafaela Lopes Freitas, Sandra Oliveira Mendes, Ricardo Sousa, Mariana Lobo, Teresa Medeiros, João Camões, Luís Miguel Afonso, Marta Pereira

**Affiliations:** 1 Internal Medicine Service, Hospital Pedro Hispano, Matosinhos, PRT; 2 Intensive Care Unit, Hospital Pedro Hispano, Matosinhos, PRT

**Keywords:** abdominal compartment syndrome, adverse effects, antipsychotics, gastrointestinal hypomotility, schizophrenia

## Abstract

Gastrointestinal hypomotility (GIH) represents a significant yet underrecognized adverse effect of antipsychotic medications that can lead to severe complications.

We present the case of a 37-year-old male with schizophrenia on first- and second-generation antipsychotics who presented to the emergency department with acute diffuse abdominal pain. Physical examination revealed a distended, silent, and diffusely tender abdomen along with compromised ventilatory mechanics and signs of peripheral hypoperfusion. Laboratory evaluation demonstrated elevated inflammatory markers, acute kidney injury, and hyperlactatemia. Abdominal computed tomography revealed significant small and large bowel distention without mechanical obstruction and a collapsed inferior vena cava.

Despite initial treatment with laxatives, the patient's clinical condition rapidly deteriorated into a peri-arrest state requiring emergency department admission. Under the working diagnosis of obstructive shock secondary to abdominal compartment syndrome, the patient underwent emergent decompressive laparotomy with immediate hemodynamic and respiratory improvement. Intraoperative findings revealed markedly distended small and large bowels with evidence of colonic unviability, necessitating total colectomy. Following a comprehensive evaluation, antipsychotic-induced GIH was determined to be the most probable underlying pathophysiological mechanism.

This case illustrates a severe manifestation of antipsychotic-induced GIH and emphasizes the critical importance of prevention strategies and early recognition of this potentially life-threatening condition.

## Introduction

Gastrointestinal hypomotility (GIH) is a well-known comorbidity among patients with mental health disorders. It encompasses a range of clinical presentations, including gastroparesis, adynamic ileus and colonic pseudo-obstruction, all of which significantly impact patients' quality of life, regardless of their severity. The most recognized clinical expression of GIH in this population of patients is constipation, a condition which seems to affect up to two-thirds of specific subpopulations of psychiatric patients [1]. The underlying causes of GIH in these patients are multifactorial, involving: (1) Medication-related factors, namely first- and second-generation antipsychotics [2,3] (with further increased risk in patients on two or more antipsychotics [4]), tricyclic antidepressants [5], and mood stabilizers [6]; (2) Lifestyle-related factors, such as physical inactivity (a common occurrence in severe mental illness and institutional settings [7]) and poor dietary habits (low fiber intake and inadequate hydration) [8]; (3) Disease-related factors, such as the negative symptoms [2] and overall severity [4] of schizophrenia (which may lead to reduced awareness of bodily needs or reduced motivation for self-care) as well as cognitive impairment (that may affect the recognition of the need to defecate or the ability to communicate symptoms) [3]; (4) Comorbid conditions, namely diabetes [9]. 

Despite its frequent association with antipsychotic use, GIH remains an under-recognized and often dismissed side effect of these drugs, resulting in diagnostic and therapeutic inertia and potentially leading to dangerous consequences. This oversight stems from several factors: (1) patients' limited ability to report symptoms due to cognitive impairment or sedation [3]; (2) a historical lack of attention to this adverse effect in the reports of drug regulation agencies [10]; and (3) inadequate awareness among healthcare professionals [11,12]. Given the frequent use of antipsychotics in clinical practice, clinicians must be able to prevent, recognize, and manage this complication. To illustrate the severity of antipsychotic-induced GIH and to raise awareness of this association, we present a case of a patient with schizophrenia who developed a life-threatening form of this condition. 

## Case presentation

A 37-year-old institutionalized male with schizophrenia presented to the emergency department (ED) with a five-day history of diffuse abdominal pain. Due to patient's cognitive impairment and after thorough inquire with the institution's staff no further history was made available.

The patient's medical history was notable for schizophrenia diagnosed on the second decade of life and under long-term treatment with first-generation antipsychotics (haloperidol decanoate 100 milligrams monthly, chlorpromazine 475 milligrams daily), second-generation antipsychotics (clozapine 400 milligrams daily, olanzapine 15 milligrams daily, amisulpride 600 milligrams daily), a benzodiazepine (flurazepam 15 milligrams daily), and an antiepileptic (valproic acid 1500 milligrams daily). The patient had a history of five ED visits over the preceding three years for episodic abdominal pain interpreted in the context of chronic constipation. During these encounters, mechanical obstruction was ruled out through imaging studies; however, no comprehensive investigation for alternative etiologies was pursued. Following each ED visit, the patient was discharged with modifications to his laxative regimen and dietary recommendations.

On examination, the patient was emaciated, dehydrated, with pain grimace and no antalgic position. He showed signs of poor ventilatory mechanics with rapid (respiratory rate of 40 cycles *per* minute) and shallow breathing despite no desaturation on room air. He was tachycardic (heart rate of 125 beats *per* minute) and had a prolonged gastric capillary reperfusion time despite a mean arterial pressure of 80 mmHg. His abdomen was distended, silent on auscultation, and diffusely tender, without signs of peritoneal irritation. A digital rectal exam revealed hardened stools. His body temperature was normal. Although severe agitation limited full neurological assessment, no significant motor deficits were observed.

Laboratory tests (Table 1) revealed elevated inflammatory parameters, and acute kidney injury. Arterial blood gas analysis showed hyperlactacidemia and mild lactic acidemia. Liver function and electrolyte levels were unremarkable.

**Table 1:** Laboratory Data. ^1^ Fraction of inspired Oxygen=0.21.

Variable	Reference Range	Results on admission
Venous Blood Sample Analysis		
Hemoglobin (gram/deciliter)	13.0-18-0	17.6
Hematocrit (%)	40.0-52.0	52.7
Platelet count (per microliter)	150000-400000	213000
White-cell count (per microliter)	4000-11000	8490
Differential count (per microliter)		
Neutrophils	1300-8800	6600
Eosinophils	0-700	0
Basophils	0-200	400
Lymphocytes	1000-4800	900
Monocytes	200-1000	900
Creatinine (milligrams/deciliter)	0.7-1.3	1.5
Urea (milligrams/deciliter)	19.0-44.0	44.0
Sodium (milliequivalents/liter)	136-145	133
Potassium (milliequivalents/liter)	3.4-5.1	4.5
Aspartate aminotransferase (U/liter)	5.0-34.0	29.0
Alanine aminotransferase (U/liter)	<55.0	19.0
Alkaline phosphatase (U/liter)	40.0-150.0	87.0
Gamma-glutamyl transferase (U/liter)	<55.0	16.0
Pancreatic Amylase (U/liter)	8.0-53.0	033.0
C-reactive protein (milligrams/liter)	<5.0	112.3
Procalcitonin (nanograms/milliliter)	<0.5	5.9
Prothrombin time (seconds)	11.5-14.5	12.5
Prothrombin-time international normalized ratio	0.9-1.1	1.1
Activated partial-thromboplastin time (seconds)	29.4	42.6
Fibrinogen (gram/liter)	2.38-4.98	2.18
Arterial Blood Gas Analysis^1^		
pH	7.35-7.45	7.34
Carbon dioxide (mmHg)	35-40	34
Oxygen (mmHg)	75-100	84
Bicarbonate (mmol/liter)	22.0-26.0	19.7
Lactic acid (mmol/liter)	0.5-2.0	6.8
Glucose (milligrams/deciliter)	80-120	139

**Figure 1:**
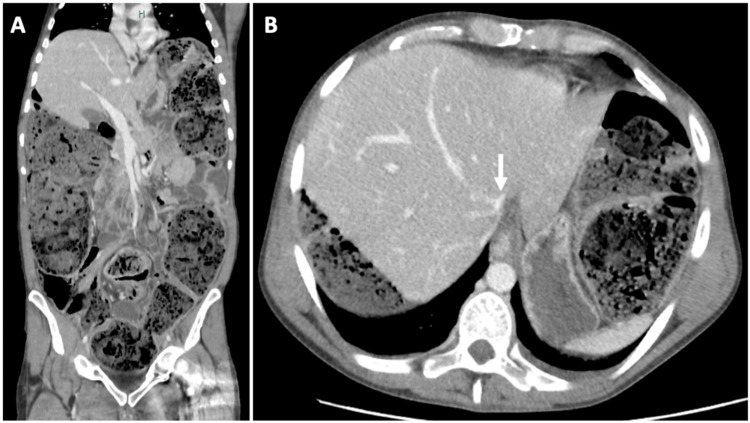
Admission abdominal-pelvic computer tomography with intravenous contrast. (A) Coronal section displaying diffusely distended large and small bowel with luminal content, with upward displacement of the diaphragm. (B) Cross sectional imaging of the upper abdominal cavity displaying a collapsed inferior vena cava (white arrow).

Contrast-enhanced abdominal CT documented a diffusely distended large bowel (right hemicolon diameter of 88mm) with fecal content and small bowel dilatation with liquid stasis causing upward dislocation of the diaphragm and compression of the inferior vena cava. No signs of mechanical bowel obstruction, ischemia, perforation, or peritonitis were noted (Figure 1A, 1B).

A diagnosis of colonic pseudo-obstruction (i.e. Ogilvie syndrome) was initially assumed, and oral laxatives and enemas were administered. Within a few hours, while in the ED, the patient's condition deteriorated into a peri-arrest state and he was admitted to the emergency room. There, he exhibited signs of shock with depressed level of consciousness, oliguria, and impending ventilatory failure. Reassessment of the case led to two working diagnostic hypotheses for the patient's clinical deterioration: obstructive shock secondary to abdominal compartment syndrome (ACS) and/or septic shock secondary to intestinal bacteria translocation, both of them developed in the context of Ogilvie Syndrome.

Immediate ACS medical management was started with vasopressors titrated to a mean arterial pressure of >80mmHg (60mmHg plus at least 20mmHg of estimated intra-abdominal pressure), sedation and neuromuscular blockade, mechanical ventilatory support with the low positive end expiratory pressure and plateau pressure as well as gastric and bladder catheterization for decompression. After multidisciplinary discussion with the surgical and intensive care teams, emergent decompressive laparotomy was performed, resulting in immediate hemodynamic and respiratory improvement, favoring the obstruction mechanism as the main pathophysiologic contributor to the shock state. Intraoperative findings confirmed massive large bowel distension without mechanical obstruction, ischemia, or perforation. Given the history of chronic GIH and the appearance of the colon, irreversible bowel dysfunction was suspected and total colectomy with terminal ileostomy was performed.

The first postoperative week was complicated by persistent GIH, manifesting as ileus. Repeated abdominal CT excluded surgical complications. A comprehensive metabolic and endocrine evaluation found no alternative causes for GIH apart from prolonged antipsychotic use, with post-surgical GIH persistence probably secondary to postoperative ileus. 

A multimodal treatment approach to GIH was implemented in the postoperative period, including initial parenteral nutrition followed by early and slowly progressive oral feeding, in parallel with strict volume management and early patient mobilization. Synchronously, a fixed regimen of metoclopramide in combination with erythromycin and oral laxatives as well as on-demand neostigmine was administered. The Psychiatry team was involved in the early management of the patient - a careful revision of the patient’s psychiatric medication was undertaken, minimizing the use of GIH-inducing agents. He was restarted on olanzapine, haloperidol decanoate, flurazepam, and valproic acid, achieving stable psychiatric control. Clozapine, chlorpromazine and amisulpride were discontinued.

With the above-mentioned measures, the patient's clinical condition improved and he was discharged after 17 days of hospitalization, tolerating a full oral diet and with a regularly functioning ileostomy on a fixed regimen of oral prokinetics and laxatives. 

Following his discharge, the patient continued to receive medical and psychiatric care at his residential institution. No further ED or admission episodes were recorded up to the present date.

## Discussion

The mechanism of antipsychotic-induced GIH remains unclear but is thought to involve antagonism of cholinergic, dopaminergic, histaminergic and serotonergic receptors [13]. Both typical and atypical antipsychotics pose a risk, with clozapine being the most frequently implicated. A dose-response relationship has yet to be definitively established [10]. The risk may increase with co-administration of other GIH-inducing drugs, such as antidepressants, opiates, and antihistamines.

The pathophysiology of antipsychotics-induced GH has been better characterized for clozapine. Its clinical expression consists of multi-regional digestive tract hypomotility with delayed gastric emptying and delayed small and large bowel transit [14]. Symptomatic patients may experience a spectrum of severity, ranging from mild to life-threatening complications of constipation such as bowel ischemia, perforation, intestinal bacterial translocation, and ACS [10], as demonstrated in our case. Pharmacovigilance data estimate 20-90 severe GIH cases per 10,000 clozapine users [10,15-17], with a fatality rate of approximately 18% [10,18]. Importantly, a significant proportion of patients under antipsychotic treatment with objectively documented delayed gastrointestinal transit report no symptoms [13,19-20], which can lead to dangerous diagnostic delays culminating in irreversible bowel dysfunction and life-threatening complications as seen in the present case. This situation may stem from reduced pain sensitivity in some psychiatric conditions such as schizophrenia [21], impaired symptom communication in this specific population [22], and the sedative and anti-serotonergic effects of antipsychotics, which can suppress visceral nociception [9]. Additionally, social isolation and disparities in healthcare access among patients with mental health disorders [9] compounded by the lack of awareness of this antipsychotic side effect among healthcare professionals [11,12] further contribute to diagnostic delays.

Given the frequent silent nature of antipsychotic-induced GIH, standardized preventive strategies should be implemented. A protocol for clozapine users has been proposed [3], emphasizing (1) the optimization of modifiable risk factors through careful attention to hydration, fiber intake, and physical activity as well as by minimizing the co-prescription of other drugs with GIH potential; (2) routine prophylactic laxative use; (3) active monitoring of gastrointestinal symptoms via stool diaries; and (4) patient and caregiver education on red flags warranting urgent evaluation. Whether these measures improve outcomes for other antipsychotics remains to be determined.

## Conclusions

This case highlights the severe and often overlooked consequences of GIH in patients with psychiatric conditions. GIH in this population of patients is frequently a multifactorial process, with potentially modifiable risk factors (namely antipsychotics) which creates opportunities for prevention.

Raising awareness of this issue among healthcare providers represents a critical step towards reducing its associated morbidity and mortality. It is the authors' opinion that systematic evaluation of gastrointestinal function and implementation of standardized management protocols encompassing both pharmacological and non-pharmacological interventions for GIH should be established as auditable quality-of-care indicators for patients with psychiatric illness. The integration of such a structured approach into routine clinical practice would facilitate earlier detection, more effective management, and, perhaps, ultimately improved outcomes for this vulnerable patient population.
